# Annotations

**Published:** 1887-09-03

**Authors:** 


### Annotations.

**The Queen's Fund and the Pension Fund.**

Several letters from nurses and others have reached us since the announcement appeared in the daily papers that her Majesty the Queen had made known her intention of devoting a portion of the Women's Jubilee Offering to the interests of sick-nurses. It would be difficult to imagine any nobler or more necessary use to which the Fund or a portion of it could be applied. No one probably knows better than the Queen what care is. A multiplication of various cares is very disabling to the worker and very injurious to the health. People who have to perform merely perfunctory services may do their work moderately well, even though their mind and thoughts be elsewhere. But the work of the sick-nurse cannot be done perfunctorily. It must be done with her whole heart and soul in her own possession at the moment. The patient needs to feel that the nurse's time and attention are wholly given to him, and at the moment, to him alone. But this cannot be the case if the nurse have many private embarrassments. One such embarrassment most nurses must frequently have, and that is the ever-present consciousness that, if they fall ill, they have no resource of any kind except that which charity may bestow: no sick-fund: no retiring pension: no prospect in the future except that of charity, poverty, or pauperism. That being so, their work must often suffer from the intruding presence of black care. A Sick and a Pension Fund would be to them almost indescribable blessings. No efforts shall be wanting on the part of the conductors of this Journal to make just representations in the proper quarter. If such representations should be successful, it may be our happiness to make known to the hospital world within this Jubilee year that the National Pension Fund is firmly established on a secure and permanent basis.

**Twenty Thousand Hospital Supporters.**

A vast concourse of people—20,000, it is said—assembled at Holbeck on Saturday to celebrate the second anniversay festival of the Holbeck, New Wortley and District Infirmary. To benighted Londoners it may be necessary to explain that New Wortley and Holbeck are suburbs of Leeds. The Yorkshire people, and especially the dwellers in the West Riding, possess a wholesome heartiness, a kind of local perfervidum, which is altogether admirable: and they could not have selected a better occasion for the display of their enthusiasm than the Hospital Festival. The word "Festival" in this connection is somewhat new. The festivities at Holbeck seem to have been in the highest degree successful, and to have aroused in the inhabitants of the district a genuine intensity of sympathy with the Infirmary and its work. We call special attention to this particular event because it is of a character which is quite strange to London and some other large towns. It is not possible to imagine a better opportunity for instructing the people, awakening interest in, and deepening sympathy with hospitals, than such an occasion as the Holbeck Festival. Everyone who attended it knew that he was going to a hospital demonstration. Each man, woman, and child went with an open and appreciative mind to hear what could be said on behalf of the institution. The knowledge imparted was associated with music, games, and every variety of pleasant circumstance. One such celebration, well carried out on a favourable day, would do more to convert the working-classes into hospital supporters than a hundred lectures or ten thousand printed appeals. So much everyone will admit. Why, then, we ask, does no London hospital or association of hospital secretaries or managers organise a similar *fête* on behalf of the Metropolitan Medical Charities? All that has been done up to the present time is the getting-up of a dozen ill-attended meetings, which nobody cared about, and which for the most part were as flat as ditch-water. Is it jealousy, or stupidity, or laziness, or a pitiful affectation of superiority to the common people, which animates London managers? We commend to them the example of the twenty thousand at Holbeck, and suggest a careful consideration of the event.

**Working-men and their Manners.**

It is not our custom in this Journal to regard working-men as belonging to a different order of beings from other men. On the contrary, we always insist that they should have their place in hospital administration, and affairs generally coming within our special purview, precisely the same as any other class. It is therefore with sincere regret that we find the *Newcastle Chronicle* reporting the proceedings of a meeting of railway men held in support of the Gateshead Infirmary in the following terms: "A rather heated discussion ensued at this point. The chairman said he had never presided over a more disorderly meeting; it was a disgrace to them. He threatened to leave the chair, and stated that it would be some time before he again presided over a meeting of railway men." That is decidedly bad. The men certainly ought to have known better. Most people think that the House of Commons, or at any rate a political meeting, is the only place at which persons of differing sentiments should be permitted to "fall out, and scratch and fight." We know, on the authority of Dr. Watts, that "birds in their little nests agree," and railway men at a temperance hall, and especially when discussing hospital questions, should think it quite bad form to break each other's heads. One small modicum of consolation may be extracted from the melancholy occurrence. It is evident the men were thoroughly interested in the subject or they would not have quarrelled. Perhaps the *Newcastle Chronicle* was unnecessarily fussy in reporting the facts about the "storm in the teacup." The *Leader* reported the meeting *minus* the squabble. Nobody would have been any the worse if the *Chronicle* had done the same.

